# RING finger protein TOPORS modulates the expression of tumor suppressor *SMAR1* in colorectal cancer via the TLR4‐TRIF pathway

**DOI:** 10.1002/1878-0261.13126

**Published:** 2022-02-05

**Authors:** Priyanka Firmal, Vibhuti Kumar Shah, Richa Pant, Samit Chattopadhyay

**Affiliations:** ^1^ National Centre for Cell Science Pune India; ^2^ Department of Biological Sciences BITS Pilani, K. K. Birla Goa Campus India; ^3^ Indian Institute of Chemical Biology Kolkata India

**Keywords:** LPS, SMAR1, STAT3, TAM, TLR4, TOPORS

## Abstract

TOP1‐binding arginine/serine‐rich protein (TOPORS), a really interesting new gene finger protein, has the ability to bind to a palindromic consensus DNA sequence that enables it to function as a potential transcriptional regulator. However, its role in regulating the transcription of cancer‐associated genes is yet to be explored. As Toll‐like receptor 4 (TLR4) agonists are known to regress solid tumors, we observed that lipopolysaccharide (LPS) induces TOPORS via a TLR4‐TIR domain‐containing adapter‐inducing interferon‐β‐dependent pathway, which in turn modulates the transcription of tumor suppressor scaffold/matrix attachment region‐binding protein 1 (*SMAR1*, also known as *BANP*). ChIP analysis showed that TOPORS binds on the *SMAR1* promoter and its occupancy increases upon LPS treatment. A previous study from our laboratory revealed that SMAR1 acts as a repressor of signal transducer and activator of transcription 3 (*STAT3*) transcription. Tumor growth, as well as tumor‐associated macrophage polarization, depends on the status of the STAT1:STAT3 ratio. LPS‐induced SMAR1 expression decreases STAT3 expression and also skews the macrophage polarization toward M1 phenotype. In contrast, LPS failed to polarize tumor‐associated macrophages to M1 phenotype in a *SMAR1‐*silenced condition, which shows the involvement of SMAR1 in dictating the fate of colorectal cancer progression. Identification of the molecular mechanism behind LPS‐mediated tumor regression would be crucial for designing cancer treatment strategies involving bacterial components.

## Graphical abstract

Lipopolysaccharide (LPS)‐induced expression of zinc finger protein TOPORS upregulates the expression of the tumor suppressor scaffold/matrix attachment region‐binding protein 1 (SMAR1) through Toll‐like receptor 4–MyD88‐independent pathway. SMAR1 represses the transcription of *STAT3*, hence shifting tumor‐associated macrophages toward an anticancer M1 profile. Decreased expression of SMAR1 is associated with poor prognosis of colon cancer. Therefore, enhancement of SMAR1 could improve the overall outcome of colorectal cancer.

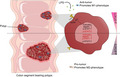

**Abbreviations**
FACS: fluorescence‐activated cell sorting
IFN: interferon
IL: interleukin
IRF: IFN regulatory factor
IRF3: interferon regulatory transcription factor 3
LPS: lipopolysaccharide
RING: really interesting new gene
SMAR1: scaffold/matrix attachment region‐binding protein 1
STAT: signal transducer and activator of transcription
TAM: tumor‐associated macrophage
TLR4: Toll‐like receptor 4
TOPORS: TOP1‐binding arginine/serine‐rich protein
TRIF: TIR domain‐containing adapter‐inducing interferon‐β
TSS: transcription start site

## 1 Introduction

Colorectal cancer (CRC) is one of the most frequent cancer and a leading cause of cancer‐associated deaths worldwide [1]. It arises due to the accumulation of various genetic and epigenetic chromatin modifications that results in the inactivation of several important tumor suppressor genes and reactivation of oncogenes [2, 3, 4, 5]. The progression of CRC is a multistep phenomenon that initiates with a somatic driver mutation followed by the development of epithelial adenoma that finally takes shape of a highly malignant and invasive adenocarcinoma. Taking into consideration the critical role played by epigenetic modifications in initiating CRC, it has been observed that nearly 95% sporadic cases, that is, people affected by CRC, have no hereditary links to disease development. Therefore, maintenance of an appropriate chromatin architecture is of utmost importance. Microbial pathogen‐associated molecular pattern (PAMP) recognition by Toll‐like receptors (TLRs) triggers an innate immune response [6]. The rapid activation of numerous TLR‐responsive genes upon ligand stimulation is due to the involvement of extensive chromatin remodeling [7]. Initially, it was believed that TLRs are only present on the immune cells, but later, their presence was also found on the cancer cells [8, 9]. TLR ligands are also known to exhibit an anticancer response through various mechanisms [10]. Lipopolysaccharide (LPS) is a specific ligand of Toll‐like receptor 4 (TLR4) and is known to regress tumors by regulating the cytokine generation and recruitment of immune cells at the tumor site [10, 11, 12, 13, 14]. TLR4 is the only receptor that utilizes both myeloid differentiation primary response protein 88 (MyD88) and TIR domain‐containing adapter‐inducing interferon‐β (TRIF)‐dependent pathway for the production of proinflammatory cytokines and type I interferons (IFNs), respectively [15]. Active TLR4 signaling regulates various LPS‐inducible genes via remodeling the chromatin organization [16, 17, 18] in a gene‐specific manner; that is, the genes responsible for providing protection to the host continues to transcribe even under LPS tolerance conditions [19]. Several expression profiling studies have also shown that majority of the LPS‐inducible genes are regulated via TLR4‐TRIF‐dependent pathway [20, 21]. A recent study involving gene regulation analysis reported that most of the MyD88‐dependent genes were transcriptionally repressed under LPS tolerance condition, whereas the genes that were actively transcribing contained an IFN regulatory factor 4 (IRF4) and zinc finger motif on their promoter [22].

TOP1‐binding arginine/serine‐rich protein (TOPORS) is a nuclear really interesting new gene (RING) family zinc finger protein that displays both ubiquitination and SUMOylation functions [23]. TOPORS was found to be colocalizing with promyelocytic leukemia nuclear bodies [24], and its expression was substantially reduced in various cancers, implicating it to be a potential tumor suppressor protein [25, 26, 27, 28, 29]. A recent study has highlighted that TOPORS regulates the higher order chromatin architecture and loss of TOPORS is associated with malignant transformation of cells [30].

Scaffold/matrix attachment region‐binding protein 1 (SMAR1) is a tumor suppressor protein expression of which is drastically reduced during cancer progression [31, 32, 33]. It is also known to negatively regulate the transcription of genes that are involved in tumorigenesis [31, 34, 35]. A study from our laboratory has shown that SMAR1 represses the transcription of signal transducer and activator of transcription 3 (*STAT3*) in T cells by binding to the matrix attachment region, −229 to −478 and −660 to −840 upstream of transcription start site (TSS) sequences present on its promoter, whereas upon interleukin (IL)‐6‐mediated suppression of SMAR1 or in the SMAR1 knockdown condition, there is an enhanced expression of STAT3, which leads to the development of ulcerative colitis and hence intensifies the risk of establishment of colorectal cancer [36]. Various studies have already highlighted the involvement of IL‐6‐STAT3 signaling axis in promoting CRC [37]. Also, an adequate balance between STAT1 and STAT3 is very crucial for shaping the fate of macrophage polarization and tumor progression [38]. LPS is known to skew the macrophage polarization toward M1 phenotype, thereby creating an antitumor microenvironment [39, 40].

Herein, we wanted to explore whether TLR ligands could regulate the expression of any tumor suppressor gene apart from the induction of proinflammatory cytokines in order to immunomodulate the tumor microenvironment. We observed that LPS treatment resulted in an enhanced SMAR1 expression via the involvement of TLR4‐TRIF‐dependent pathway. *In silico* analysis revealed an increase in the occupancy of TOPORS [25, 28] on *SMAR1* promoter. TOPORS activates the transcription of genes by binding to a particular palindromic consensus sequence [41]. *SMAR1* promoter harbors the identical TOPORS binding sequence. We also observed that LPS‐induced SMAR1 promotes M1 macrophage polarization by modulating the expression of STAT3. This study reveals an alternate molecular mechanism behind LPS‐mediated tumor regression that involves TOPORS‐regulated fine‐tuning of tumor suppressor SMAR1, which dictates the ultimate fate of colorectal cancer progression.

## 2 Materials and methods

### 2.1 Cell culture and reagents

HCT116, HT29, HEK293T, MCF7, and CT26 cells were cultured in Dulbecco's Modified Eagle Medium (DMEM). All these cell lines were maintained at 37 °C in an incubator with 5% CO_2_ and 95% relative humidity. L‐15 media were used to culture SW620 and SW480 cell line at 37 °C without CO_2_. Caco‐2 cells were cultured in MEM, whereas RPMI media were used to culture 4T1 and COLO‐205 cells. All the media were supplemented with 10% FBS, 100 U·mL^−1^ penicillin, and 100 µg·mL^−1^ streptomycin (Invitrogen, Carlsbad, CA, USA). DMEM, L‐15, MEM, and RPMI were purchased from Gibco, Thermo Fisher Scientific, Waltham, MA, USA. Except for CT26 and 4T1, all the other cell lines were obtained from Cell Repository, NCCS, Pune, India. CT26 and 4T1 cells were kindly provided by A. Bajaj, Regional Centre for Biotechnology, India, and M. Wani, National Centre for Cell Science, India, respectively. The authentication of all the cell lines was done using short tandem repeat (STR) profiling, and a 100% match was found between the tested cell lines and the ATCC STR profile database. Once revived, the cells were kept in culture for up to 10 passages. Cells were stimulated *in vitro* with LPS/Flagellin/Pam3CSK4 for 24 h prior to protein and RNA isolation. TLR4 ligand LPS [*Escherichia coli* 055:B5 (Cat. no.: L6529‐1MG)], FITC‐LPS (Cat. no.: F8666‐5MG), TLR5 ligand Flagellin (Cat. no.: SRP8029‐10UG), TLR4 inhibitor TAK‐242 (Cat. no.: 614316‐5MG), TBK1/IKKε (downstream of TRIF) inhibitor BX‐795 (Cat. no.: SML0694‐5MG), and TLR3 ligand Poly(I:C) (Cat. no.: I3036‐2MG) were procured from Sigma‐Aldrich, St. Louis, MO, USA. Anti‐TLR4‐neutralizing antibody (HTA 125; Cat. No.: 312808) was procured from BioLegend, San Diego, CA, USA. TLR1/2 ligand Pam3CSK4 (Cat. no.: sc‐202271) was obtained from Santa Cruz Biotechnology (Santa Cruz, CA, USA). MyD88 homodimerization inhibitor peptide set (Cat. no.: NBP2‐29328) was purchased from Novus Biologicals, Centennial, CO, USA. The list of all the antibodies used along with supplier details is provided in Table S1.

### 2.2 Expression vectors, siRNAs, and transfection

Full‐length TOPORS was cloned in 3X‐FLAG CMV vector (Sigma‐Aldrich) and was used for overexpression studies in HCT116 cell line. Silencer™ select siRNA against *SMAR1* (s233732), *TOPORS* (s19912), and interferon regulatory transcription factor 3 (*IRF3*; s79432) were purchased from Thermo Fisher Scientific. Silencer™ select negative siRNA (Cat. no.: 49390843) was used as control. Transfection was performed once the cells reached 70–80% confluency. Lipofectamine 2000 was used for transfecting plasmid DNA, whereas siRNA transfection was done using RNAi Max (Invitrogen) in the ratio of 1 (plasmid/siRNA) : 3 (Lipofectamine 2000/RNAi Max). The cells were cultured for 24 h after transfection and were kept for additional 24 h upon LPS treatment and under control conditions.

### 2.3 Lentiviral transduction and *in vivo* tumor model

BALB/c female mice (6–8 weeks) were acquired from animal facility at National Centre for Cell Science, India (Registration No. 7/GO/ReBi/s99/CPCSEA:09/03/1999). Experiments were performed in accordance with the CPCSEA guidelines after ethical clearance from the institutional committee (Project No. IAEC/EAF/2018/327). The mice were housed in a germ‐free condition throughout the experiment. Tumor model was generated by injecting 1 × 10^6^ control or *SMAR1* knockdown stable CT26 cells subcutaneously in the BALB/c mice. *SMAR1* knockdown stable CT26 cells were generated by transducing control or Lenti‐*SMAR1* (previously described protocol [42]) by mixing viral supernatant and culture medium in 1 : 1 ratio. Selection of stable CT26 cells was done using puromycin (1.5 μg·mL^−1^), which was purchased from Sigma‐Aldrich. Once the tumor volume reached approximately 5 mm^3^, LPS (5 µg/100 µL) or PBS (100 µL) was injected around the tumor site every alternate day for 12 days. The animals were euthanized using CO_2_, and tumors were harvested for further examination.

### 2.4 Western blotting

Harvested cells were lysed for 30 min in TNN buffer comprising of 50 mm Tris‐Cl (pH 7.5), 1% NP‐40, 150 mm NaCl, 1 mm DTT, 1 mm EDTA along with 1 mm PMSF, 1X Protease inhibitor cocktail (Pierce, Thermo Fisher Scientific, Waltham, MA, USA), and phosphatase inhibitor, 1X PhosSTOP (Sigma‐Aldrich). The protein extraction was done by centrifuging the lysed samples for 30 min at 16 000 ***g*** at 4 °C. Quantification of the protein present in the collected supernatant was performed using Bradford's reagent (Bio‐Rad, Hercules, CA, USA). After quantitation, equal amount of protein was resolved on 10% SDS/PAGE and was electrotransferred to poly(vinylidene difluoride) membrane (Millipore, Burlington, MA, USA). Blocking of membrane was done either in 5% skimmed milk or in 5% BSA (MP Biomedical, Irvine, CA, USA) to prevent any nonspecific binding. The membranes were incubated overnight with primary antibody at 4 °C. The primary antibodies used for immunoblotting were SMAR1 (Bethyl Laboratories, Montgomery, TX, USA); β‐actin and TLR4 (Santa Cruz Biotechnology); STAT1, STAT3, pSTAT1, pSTAT3, p‐p65, p65, pJNK, JNK, IRF3, p‐IRF3, and FLAG (Cell Signaling Technologies, Beverly, MA, USA); and TOPORS (Santa Cruz Biotechnology and Novus). The membranes were then incubated for 1 h with horseradish peroxidase (HRP)‐tagged species‐specific secondary antibodies at room temperature. The signal detection was done by chemiluminescence using ECL chemiluminescence substrate (Thermo Fisher Scientific). Quantitation of all the blots was done, and the values below the blot represent the fold change relative to control, which was calculated after normalization with β‐actin.

### 2.5 Co‐immunoprecipitation

Cells were lysed using TNN buffer [50 mm Tris‐Cl (pH 7.5), 1% NP‐40, 150 mm NaCl, 1 mm DTT, 1 mm EDTA, 0.5 mm PMSF, 1X Protease inhibitor (PI) cocktail, and phosphatase inhibitor (1X PhosSTOP) cocktail], and 500 µg of protein was used for a single reaction. The whole‐cell lysate was precleared with IgG and protein A/G beads for 1 h at 4 °C. Next, the precleared cell lysate was incubated overnight with specific antibody, keeping it on constant (7 r.p.m.) rotation at 4 °C. The immunoprecipitant complex was then pulled down using A/G beads (Invitrogen) at 4 °C. After thoroughly washing the beads with IP buffer, proteins bound to A/G beads were eluted using 2X SDS dye and were detected by western blotting.

### 2.6 Real‐Time PCR

Total RNA extraction from cultured cells was done using TRIzol reagent (Invitrogen) according to the prescribed protocol, and cDNA was synthesized from 2 µg RNA using M‐MLV RT Master Mix (Invitrogen). The cDNAs were amplified with iQ SYBR Green Real‐Time PCR Supermix (Bio‐Rad Laboratories). The mRNA transcript expression of the gene of interest was normalized in accordance with the housekeeping gene, that is, *18S rRNA*. The calculation of relative fold change of the test sample was done with respect to the control condition utilizing the formula 2^(−ΔΔCt)^, where (−ΔΔCt) = (Ct control − Ct target). The complete primer list and their sequences are provided in Table S2.

### 2.7 *In silico* analysis of *SMAR1* promoter for luciferase reporter assay

Transcription factor binding sites on *SMAR1* promoter region were analyzed using online available TRANSFAC database [43] in order to understand the fundamentals of transcriptional regulation of *SMAR1* upon LPS treatment. Transcription factor LUN1/TOPORS was showing the maximum binding score. After thoroughly reviewing the literature, we observed that *SMAR1* promoter harbors the exact consensus sequence on which TOPORS is known to bind. Hence, transcription factor TOPORS was shortlisted for further *in vitro* analysis.

### 2.8 *SMAR1* promoter cloning and dual‐luciferase reporter assay

Sequence of *SMAR1* promoter was collected from Eukaryotic Promoter Database (EPD) [44]. The selection of minimal promoter region for cloning [713bp (upstream of TSS)] was based on the presence of TOPORS binding consensus sequence [41] within that region, along with other regulatory elements such TATA box. Next, the selected region was amplified by PCR from human genomic DNA using promoter‐specific forward primer (KpnI restriction site) 5′‐GGGGTACCTTTTGCCACGAAGTAACCCA‐3′ and reverse primer (HindIII restriction site) 5′‐CCCAAGCTTTGTGCGTTTGTGGGTAATCA‐3′ (IDT, Coralville, IA, USA). It was then inserted in pGL3 basic luciferase reporter vector (Promega, Madison, WI, USA) between KpnI and HindIII restriction sites (New England Biolabs, Ipswich, MA, USA), and the cloned vector is mentioned as *SMAR1* promoter construct in this study. Control pGL3 basic vector (2.0 µg·mL^−1^) or *SMAR1* promoter construct (2.0 µg·mL^−1^) was transfected in HCT116 cells along with internal control Renilla luciferase vector (200 ng·mL^−1^). The cells were also co‐transfected with negative siControl and si*TOPORS* (10 pm) wherever required. After 24 h of transfection, cells were treated with TLR4 inhibitor (TAK‐242) 1 h prior to LPS (1 µg·mL^−1^) stimulation if needed and were harvested 24 h post‐treatment. Dual‐luciferase assay was performed as per the manufacturer's instructions (Promega). Modulus II Multi‐Mode Plate Reader from Turner Biosystems (Promega) was used to measure the relative light units. Differences in transfection efficiencies of firefly luciferase activity were corrected with Renilla activity.

### 2.9 ChIP

Cross‐linking of approximately 1 × 10^6^ HCT116 cells was done using 1% formaldehyde for 15 min at room temperature. Cells were then thoroughly washed with 1X PBS, and 125 mm glycine was added for 2 min to quench the cross‐linking reaction. Next, the cells were harvested by centrifugation at 8000 ***g***   for 5 min at 4 °C. Cells were then lysed in SDS lysis buffer containing PI cocktail for 10 min followed by shearing of genomic DNA by sonicating the lysate in order to obtain the DNA fragment of ˜ 500 bp. After centrifugation, 20 μL of the supernatant containing sheared chromatin was kept aside as input. The preclearance of sonicated chromatin was done for 1 h by adding 10 μL of Salmon sperm DNA/Protein A agarose beads (EMD Millipore Corp., Burlington, MA, USA) at 4 °C. Beads were separated by centrifugation at 1400 ***g*** for 1 min, and 2 μg of TOPORS (Novus), H3K9ac and H3K27me3 (Cell Signaling Technologies) antibody was added to the respective tube containing diluted supernatant having 10 μg of chromatin, which was then incubated overnight at 4 °C. Salmon sperm DNA/Protein A agarose beads (15 μL) were added for another 1 h to precipitate the antibody–DNA complex. The beads were collected by gentle centrifugation at 1400 ***g***   for 1 min and were then washed sequentially with low salt, high salt, LiCl, and TE buffer by rotating at 14 r.p.m. for 5 min followed by bead collection via centrifugation at 1400 ***g***  for 1 min each. Elution of histone complexes from antibody was done using freshly prepared elution buffer. Reverse cross‐linking was performed in all the samples, as well as the input using 5 m NaCl at 65 °C for 4 h. After this, all the proteins present in the sample and input were degraded by proteinase K treatment for 1 h at 45 °C. DNA extraction was performed by phenol/chloroform/isoamyl alcohol, and quantitative PCR was done using *SMAR1* promoter‐specific primers, that is, forward: TTATTGGCAAAAGGGAGTTGGG, reverse: CGAGGCAGCTATTTTCACTGG.

### 2.10 Immunocytochemistry and immunohistochemistry

Cells were seeded in the chamber slides and were treated with LPS (1 µg·mL^−1^) for 24 h. Fixation of cells was done using 4% paraformaldehyde for 10 min at room temperature. The cells were then thoroughly washed thrice with 1X PBS and permeabilized for 10 min using 0.1% Triton X‐100. The blocking of cells was done in 5% FBS for 1 h followed by overnight incubation in desired primary antibody at 4 °C. Next, fluorescent‐labeled secondary antibody cocktail was added to the cells for 1 h and incubation was done in a dark place at room temperature. The slides were visualized in Olympus FV3000 CLSM confocal microscope (Olympus, Tokyo, Japan). Tumor sections were paraffin‐embedded for carrying out immunohistochemistry (IHC) and hematoxylin and eosin (H&E) staining. Observation of H&E slides was done at 20× magnification using a Nikon light microscope (Nikon, Tokyo, Japan). For IHC, the slides were deparaffinized and rehydrated briefly. After antigen retrieval, the permeabilization of tissue sections was done using 0.1% Triton X‐100 for 15 min. Overnight incubation was done after adding primary antibody against SMAR1 (1 : 500) and TOPORS (1 : 200) at 4 °C. Incubation of tissue sections in secondary antibody cocktail (Life Technologies, Carlsbad, CA, USA) was done at room temperature for 1 h. The tissue sections were then mounted in fluoroshield media with DAPI (Sigma‐Aldrich) and were visualized in Olympus FV3000 CLSM Confocal microscope at 63× magnification.

### 2.11 Macrophage isolation and FACS analysis

Peritoneal macrophages were isolated from BALB/c mice and cultured in RPMI medium. After they attained morphology, the cells were transfected with si*SMAR1* (20 pm). Conditioned media (CM) from CT26 cells (control and LPS‐treated) were added in cultured macrophages and incubated for 24 h. IFN‐β‐neutralizing antibody (Cat no.: 324001, Thermo Fisher Scientific) was also added in one of the LPS‐treated CT26 cells; later, these CM were added in the cultured macrophages to check the effect of depletion of IFN‐β on macrophage polarization. After incubation, the cells were stained with F4/80 APC, CD45R FITC, CD86‐PE, and CD 206‐PE (eBioscience, San Diego, CA, USA) antibodies and the macrophage polarization was analyzed using fluorescence‐activated cell sorting (FACS). flowjo version 7 software (BD Bioscience, San Jose, CA, USA) was used for data analysis.

### 2.12 ELISA

Conditioned media were collected from CT26 cell line upon LPS treatment at 6, 12, and 24 h. Sandwich ELISA was carried out according to the manufacturer's defined protocol to measure the levels of IFN‐β, IL‐1β, IL‐12, and TNF (R&D Systems, Minneapolis, MN, USA) in the collected CM.

### 2.13 Statistical analysis

The data are represented as mean ± standard deviation. All the *in vitro* experiments were performed three times independently. The mean of different conditions was compared using Student's *t*‐test or by one/two‐way ANOVA, followed by *post hoc* Bonferroni test wherever required. The statistical significance was accepted when *P* < 0.05. graphpad prism (v.5.01) (GraphPad, San Diego, CA, USA) was used to plot the graphs and perform all the statistical analyses.

## 3 Results

### 3.1 TLR4 agonist, neither TLR1/2 nor TLR5, upregulates SMAR1 expression

**Fig. 1:**
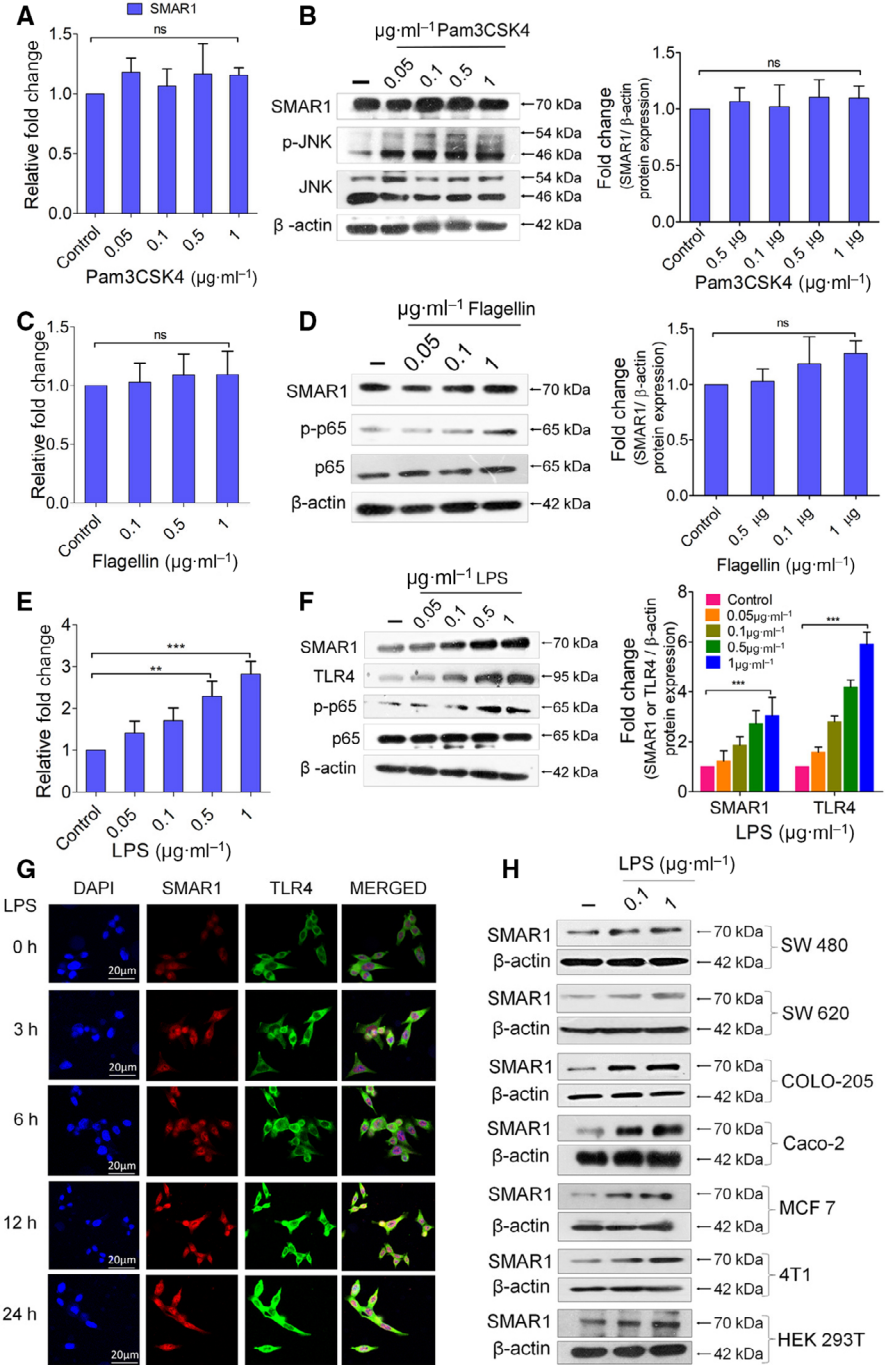
SMAR1 expression upon TLR agonist treatment. Western blot showing the expression of SMAR1 during the concentration‐dependent treatment of (A) TLR1/2 ligand, Pam3CSK4, (C) TLR5 ligand, Flagellin, and (E) TLR4 ligand, LPS, in HCT116 cell line. Total and phospho‐p65 or JNK were used as positive controls. β‐Actin was used as an endogenous loading control. The graphs represent the fold change relative to control, which was calculated after normalization with β‐actin. Real‐time quantification of *SMAR1* transcript was also carried out in a dose‐dependent manner upon (B) Pam3CSK4, (D) Flagellin, and (F) LPS treatment in HCT116 cell line. Relative fold change for all the real‐time quantitation was calculated using 18S rRNA. Confocal images representing the (G) time‐dependent expression of SMAR1(red) and TLR4 (green) under LPS (1 μg·mL^−1^) treatment condition. DAPI was used to stain the nucleus. The scale bar represents 20 μm. (H) Western blot showing SMAR1 expression upon LPS treatment in different human colon cancer cell lines of increasing grades, human breast cancer cell line, MCF 7, a mouse breast cancer cell line 4T1 along with a noncancerous cell line HEK293T. Data shown are representative of three independent experiments. Error bars indicate that all the values are mean ± SD, where **P < 0.01, ***P < 0.001 (one‐way ANOVA, Bonferroni post‐tests).

Previous studies have shown that TLR agonists have the potential to regress tumors by regulating the expression of various proinflammatory cytokines, thereby immunomodulating the tumor microenvironment [45, 46, 47, 48]. To investigate whether TLR agonists exert any effects on the expression of tumor suppressor SMAR1, HCT116 cells were stimulated with Pam3CSK4 (TLR1/2 agonist), Flagellin (TLR5 agonist), and LPS (TLR4 agonist) in a concentration‐dependent manner for 24 h. We observed that the expression of *SMAR1* transcript under Pam3CSK4 and Flagellin stimulation was changed by only 1.15‐ and 1.2‐fold, respectively, as compared to the control (Fig. 1A,C), which is also being reflected at the protein level (Fig. 1B,D). In contrast, treatment with 1 µg·mL^−1^ LPS elevated *SMAR1* expression by threefold at transcript level (Fig. 1E) that eventually enhances its protein expression (Fig. 1F). The expression of TLR4 is also known to upregulate under LPS stimulation via TLR4‐IRAK‐NF‑κB pathway [49]. An enhanced expression of TLR4 was also observed along with SMAR1 in HCT116 cells. Once activated, TLR signaling is known to trigger NFκ‐B and MAP kinase pathway [50]. In order to confirm the activation of TLR pathway upon stimulation with specific ligands, expression of positive controls, that is, total and phosphorylated p65 and JNK, was also assessed via western blotting in HCT116 cells. β‐Actin was used as an internal control. Hence, it can be concluded that enhanced SMAR1 expression is because of the increase in its transcription, as no change was observed while looking for the degradation of SMAR1 by ubiquitination pathway upon LPS stimulation (Fig. S1A). A time‐dependent study also displayed a gradual enhancement of SMAR1 expression at protein level upon increasing the LPS treatment time, which was observed to be declining at 48‐h time point that could be due to excessive cell death at both protein (Fig. S1B) and mRNA (Fig. S1C) levels. Immunofluorescence studies highlighted that TLR4 expression also increases upon LPS treatment (Fig. 1G). In order to confirm the consistency of SMAR1 expression upon LPS stimulation, cancer cell lines of different grade and origin along with HEK293T cells were induced with LPS in a concentration‐dependent manner. Indeed, LPS was able to induce SMAR1 expression in all the tested cell lines at protein level (Fig. 1H). Next, mRNA expression of *SMAR1* transcript was checked in all the tested cell lines and an increase in the *SMAR1* transcript level was observed, which was finally being reflected at its protein expression (Fig. S1D). Also, flow cytometry studies revealed an increase in the surface expression of TLR4 upon LPS stimulation (1 µg·mL^−1^) in different colorectal cancer cell lines (Fig. S1E). These findings suggest that LPS stimulation universally induce SMAR1 expression independent of its origin. Hence, it can be concluded that no other surface TLR agonist apart from LPS was able to regulate the expression of SMAR1.

### 3.2 SMAR1 expression is governed by TLR4‐TRIF‐dependent pathway

**Fig. 2:**
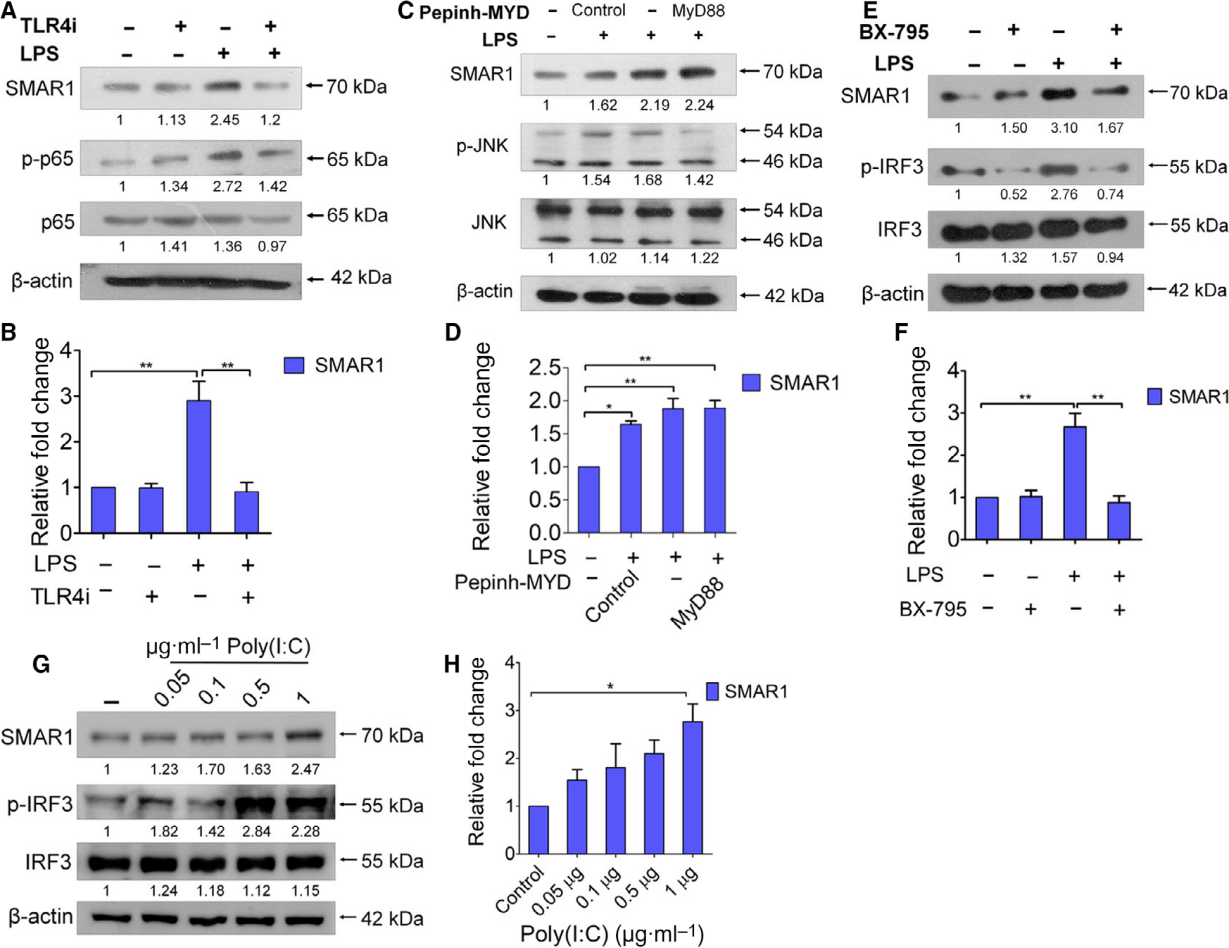
SMAR1 expression upon LPS treatment after inhibiting TLR4 signaling and its downstream pathways. Western blot analysis showing SMAR1 expression upon (A) inhibiting TLR4 pathway using TAK‐242 (5 μm) in the absence and presence of LPS (1 μg·mL^−1^) treatment in HCT116 cell line. Only LPS treatment condition was used as positive control. Total and phospho‐p65 were used as positive control (B) Real‐time analysis was carried out to check the transcript levels of *SMAR1* in HCT116 cell line upon inhibiting TLR4 pathway. Next, MyD88‐dependent pathway was inhibited using peptide inhibitor against MyD88 (Pepinh‐MYD: 10 μm). (C) SMAR1 expression was checked by western blotting, where pJNK/JNK was used as positive control to track the activation of MyD88 pathway. (D) Real‐time quantification of *SMAR1* transcript was also done upon inhibition of MyD88 pathway. TRIF‐dependent pathway was inhibited using BX‐795 (10 μm). (E) Expression of SMAR1 was observed using western blot and (F) real‐time PCR upon inhibiting TRIF‐dependent pathway. TLR3 agonist Poly(I:C) also uses TRIF‐dependent pathway to relay the downstream signals. (G) Western blot showing the expression of SMAR1 upon Poly(I:C) treatment, where the expression of p‐IRF3 was used as positive control. (H) Transcript levels of *SMAR1* after Poly(I:C) treatment in HCT116 cell line. β‐Actin was used as an endogenous loading control in all the western blots. The values below the blot represent the fold change relative to control, which was calculated after normalization with β‐actin. Relative fold change for all the real‐time quantitation was calculated using *18S rRNA*. Data shown are representative of three independent experiments. Error bars indicate that all the values are mean ± SD, where *P < 0.05 **P < 0.01(one‐way ANOVA, Bonferroni post‐tests).

TLR4 is known to trigger the innate immune response upon LPS recognition [51]. In order to confirm the involvement of TLR4 pathway in regulating SMAR1 expression, TLR4 signaling was inhibited using a specific chemical inhibitor TAK‐242 (TLR4i) and TLR4‐neutralizing antibody (HTA 125). It has been observed that LPS stimulation does not affect protein and transcript levels of SMAR1 if TLR4 signaling is abrogated (Fig. 2A,B, Fig. S2A,B). Upon ligand binding, TLR4 relays the downstream signaling via two different routes, that is, MyD88‐dependent and TRIF‐dependent pathways [6]. To further delineate the pathway, we inhibited TLR4‐MyD88 pathway using MyD88 homodimerization inhibitor peptide set, containing Pepinh‐MyD (DRQIKIWFQNRRMKWKKRDVLPGT) and Pepinh‐Control (DRQIKIWFQNRRMKWKK) (Cat. no.: NBP2‐29328) [52]. We observed that in spite of MyD88 pathway inhibition, protein and transcript levels of *SMAR1* were elevated upon LPS stimulation, confirming MyD88 pathway has no role to play in LPS‐induced SMAR1 expression (Fig. 2C,D). On the contrary, upon TLR4‐TRIF pathway inhibition using BX‐795 (Cat. no.: SML0694), LPS treatment was unable to trigger the expression of SMAR1 protein and its transcript (Fig. 2E,F). These observations strongly highlight that enhanced expression of SMAR1 by LPS stimulation utilizes TLR4‐TRIF‐dependent pathway. Apart from TLR4, TLR3 is the only PRR that signals via TLR4‐TRIF pathway. So, we checked the expression of SMAR1 upon TLR3 pathway stimulation. We found that similar to LPS, TLR3 ligand Poly(I:C) was also able to enhance the expression of SMAR1 protein and 2.5‐fold increase in its transcript level in HCT116 cells confirming the involvement of TLR4‐TRIF pathway (Fig. 2G,H). TLR4‐TRIF pathway also results in the generation of type 1 IFNs by activating transcription factor, IRF3. Silencing of *IRF3* using siRNA (10 pm) resulted in a decrease in the expression of SMAR1, implicating that SMAR1 is downstream to IRF3 in TLR4‐TRIF signaling cascade (Fig. S2C). Internalization of TLR4 happens at a later stage upon ligand binding, which is essential for TRIF‐dependent signaling to progress. We observed the LPS uptake in a time‐dependent manner using FITC‐tagged LPS by confocal microscopy. Indeed, FITC‐LPS was found to be located in the cytoplasm after few hours of the treatment (Fig. S2D). Collectively, these findings imply that TLR4‐TRIF‐dependent pathway and not MyD88‐dependent signaling is responsible for regulating SMAR1 expression.

### 3.3 TOPORS positively regulates *SMAR1* expression at transcription level

**Fig. 3:**
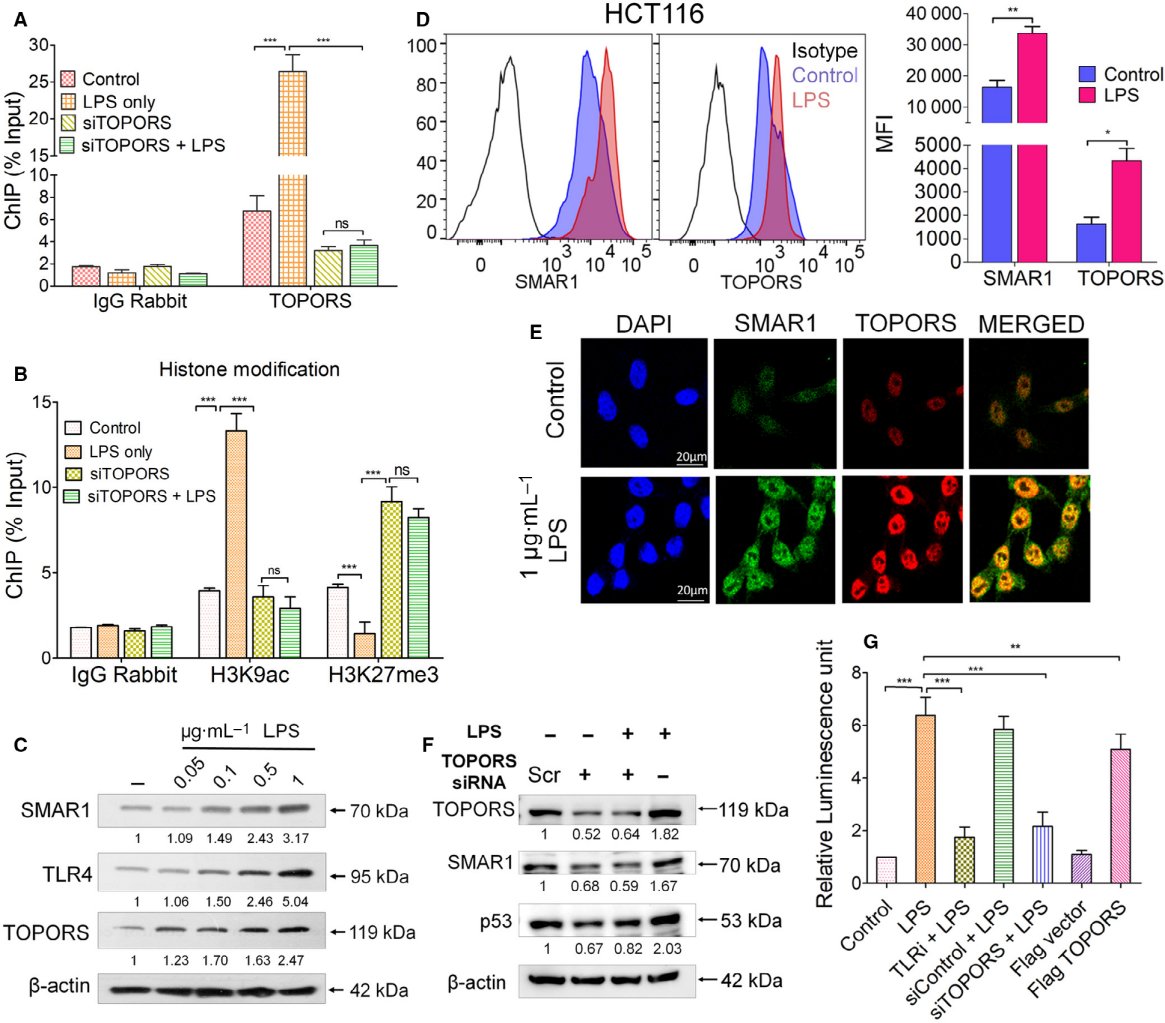
Occupancy of TOPORS increases on *SMAR1* promoter during LPS treatment. (A) ChIP of *SMAR1* promoter by TOPORS in HCT116 cell line after knocking down *TOPORS* using siRNA (10 pm) upon LPS (1 μg·mL^−1^) treatment. Histone modifications were also studied by pulling the DNA with H3K9ac and H3K27me3 mark‐specific antibodies where (B) H3K9ac and H3K27me3 marks were analyzed during LPS treatment. Representative (C) western blot showing the changes in the protein expression of TOPORS, SMAR1, and TLR4 upon LPS stimulation. (D) FACS plots along with graph showing median fluorescent intensity of SMAR1 and TOPORS in HCT116 cell line. (E) Immunofluorescence images showing the expression of TOPORS and SMAR1 in the control and LPS‐treated condition in HCT116 cell line. The scale bar represents 20 μm. Western blot representing the (F) expression of SMAR1 upon *TOPORS* silencing, where p53 was used as a positive control. *SMAR1* promoter luciferase assay was carried out in (G) HCT116 cells using SMAR1 reporter cloned in pGL3 vector. β‐Actin was used as an endogenous loading control in all the western blots. The values below the blot represent the fold change relative to control, which was calculated after normalization with β‐actin. Data shown are representative of three independent experiments. Error bars indicate that all the values are mean ± SD, where *P < 0.05, **P < 0.01, and ***P < 0.001, ns: nonsignificant (two‐way ANOVA, Bonferroni post‐tests).

In the current study, through *in silico* analysis, it has been found that *SMAR1* promoter contains an identical TOPORS binding consensus sequence (Fig. S3A). Hence, we speculated that TOPORS might have a role to play in regulating SMAR1 expression. To verify that, ChIP was performed in order to find the binding of TOPORS on *SMAR1* promoter. Interestingly, TOPORS was indeed binding on *SMAR1* promoter as its occupancy was increased by 3.5‐fold upon LPS treatment as compared to the untreated condition. Silencing of *TOPORS* using siRNA (10 pm) resulted in a decrease in immunoprecipitation of chromatin as compared to treatment condition (Fig. 3A). Next, to check whether LPS stimulation resulted in any changes in the histone modification pattern, a ChIP analysis was carried out and it has been observed that the status of H3K9ac mark increases by 3.5‐fold, whereas H3K27me3 on *SMAR1* promoter decreases by twofold under the influence of LPS. On the contrary, *TOPORS* silencing reversed the histone marks (Fig. 3B). We, for the first time, observed that the along with SMAR1, expression of TOPORS (LUN1) is also being enhanced upon LPS treatment in HCT116 cell line (Fig. 3C and Fig. S3B), which was also confirmed by FACS analysis and immunofluorescence studies (Fig. 3D,E) in HCT116 and SW620 cell line (Fig. S3C,D). Further, to find the effect of TOPORS on SMAR1 expression, *TOPORS* was silenced using siRNA (10 pm). A reduction in SMAR1 expression has been observed, which was not replenished even upon LPS treatment, suggesting that the presence of TOPORS is critical for SMAR1 expression (Fig. 3F and Fig. S3E). As TOPORS is already known to positively regulate the expression of p53, p53 was used as a positive control. Overexpression of TOPORS in HCT116 cells resulted in an enhanced SMAR1 expression even in the absence of LPS, which implies that LPS induces SMAR1 via TOPORS (Fig. S3F). We also observed a sixfold increase in the relative luciferase activity of *SMAR1* promoter region harboring TOPORS binding site upon LPS treatment as compared to the control condition. On the contrary, inhibition of TLR4 pathway and silencing of *TOPORS* hindered the promoter activity even upon LPS stimulation, whereas overexpression of TOPORS alone was able to activate *SMAR1* promoter (Fig. 3G). Hence, it can be speculated that TOPORS positively regulates SMAR1 expression via regulating its transcription upon LPS treatment.

### 3.4 SMAR1 favors M1 macrophage phenotype by regulating STAT3 expression

**Fig. 4:**
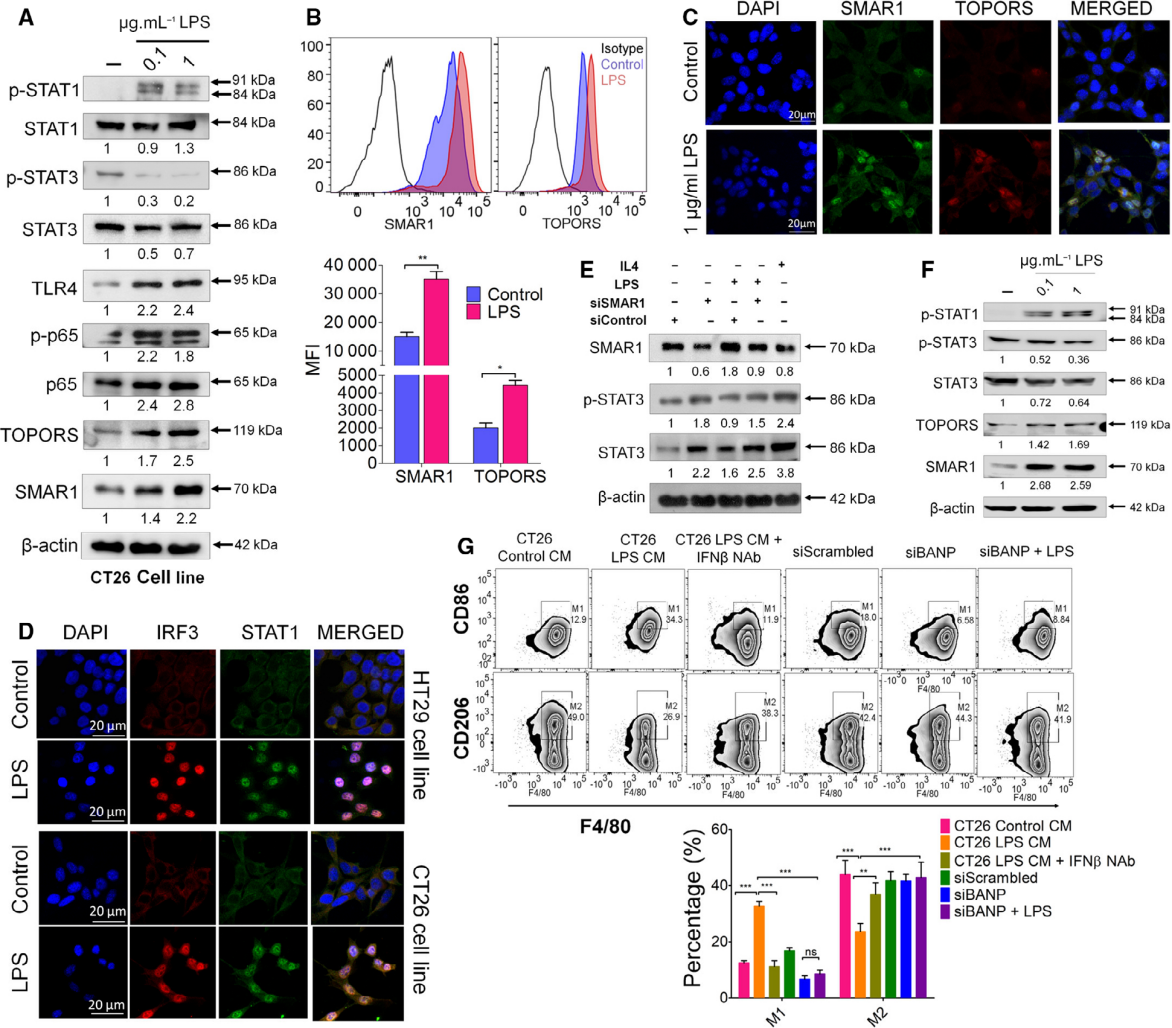
SMAR1‐mediated regulation of STAT3 upon LPS treatment. Western blot showing reduced STAT3 expression in (A) CT26 cell line upon LPS treatment. SMAR1 and TOPORS expressions increase in CT26 (mouse colon cancer) cell line as well. (B) Representative FACS plot along with graph showing median fluorescent intensity. (C) Immunofluorescence images depicting the expression of SMAR1 and TOPORS in CT26 cell line. (D) Confocal images showing the expression of IRF3 and STAT1 under LPS‐treated conditions in HT29 and CT26 cell lines. The scale bar represents 20 μm. Western blot representing the (E) expression status of pSTAT3 during SMAR1 knockout conditions in peritoneal macrophages (*n* = 6) with or without LPS treatment and (F) expression of SMAR1, TOPORS, and STATs in peritoneal macrophages during dose‐dependent LPS treatment. (G) Representative FACS plots of M1 (F4/80^+^ CD 86^+^)‐ and M2 (F4/80^+^ CD 206^+^)‐polarized peritoneal macrophage (*n* = 12) using CM collected from CT26 cells with or without LPS. β‐Actin was used as an endogenous loading control in all the western blots. The values below the blot represent the fold change relative to control, which was calculated after normalization with β‐actin. Data shown are representative of three independent experiments. Error bars indicate that all the values are mean ± SD, where *P < 0.05, **P < 0.01, ad ***P < 0.001 (two‐way ANOVA, Bonferroni post‐tests).

We have reported in one of our previous study that SMAR1 binds at the MAR binding region on *STAT3* promoter and thereby represses its transcription in T cells [36]. Keeping in mind the importance of STAT1 and STAT3 balance during tumorigenesis, the levels of STAT1 and STAT3 were checked in CT26 and HT29 cell line 24 h post‐LPS treatment and a decrease in STAT3 expression was observed. Additionally, there was an increase in the expression of pSTAT1 along with SMAR1 and TOPORS in the presence of LPS (Fig. 4A and Fig. S4A). Next, a time‐dependent study was carried out and it has been observed that as the expression of SMAR1 increases, STAT3 expression gets downregulated under LPS stimulation (Fig. S4B). Increase in the intracellular expression of SMAR1 and TOPORS was further confirmed by FACS analysis and immunofluorescence imaging (Fig. 4B,C). Nuclear translocation of IRF3 and STAT1 was also observed in the cells treated with LPS (Fig. 4D). To further confirm the involvement of SMAR1 in regulating STAT3 expression, we silenced *SMAR1* using siRNA (20 pm) and observed that under SMAR1‐deficit conditions, the expression of STAT3 increases (Fig. 4E and Fig. S4C). Confocal data also strengthened the fact that pSTAT1 and pSTAT3, as well as SMAR1 and STAT3, have inverse expression pattern (Fig. S4D,E). Next, ELISA was performed in order to check the cytokine levels in the CM collected from CT26 cells treated with LPS at different time intervals. We observed a distinct cytokine profile in the CM collected from LPS‐treated cells and the untreated condition (Fig. S4E). Peritoneal macrophages incubated with LPS displayed an enhanced expression of SMAR1, TOPORS, and p‐STAT (Fig. 4F). There was a fourfold increase in the transcript levels of *SMAR1* in the presence of LPS along with an elevated transcription of proinflammatory cytokines (Fig. S4F). As SMAR1 increases, the expression of STAT3 decreases, and under low STAT3 conditions, macrophage polarization is skewed toward M1 phenotype. To investigate the functional implication of these proinflammatory cytokines in tumor microenvironment, the CM collected from CT26 cells was used to polarize the peritoneal macrophages. It has been observed that when macrophages were cultured with LPS‐treated CM, the population of M1 macrophages was 2.5% more as compared to the control condition, whereas LPS‐treated CM with depleted IFN‐β failed to polarize the naïve macrophage to M1 phenotype; rather, these conditions promoted M2 macrophages. Similarly, CM collected from *SMAR1*‐silenced cells stimulated with LPS also failed to polarize the macrophage to M1 profile (Fig. 4G). This suggests that SMAR1 plays a crucial role in macrophage polarization.

### 3.5 LPS‐induced SMAR1 regresses tumors via altering TAMs to M1 phenotype

**Fig. 5:**
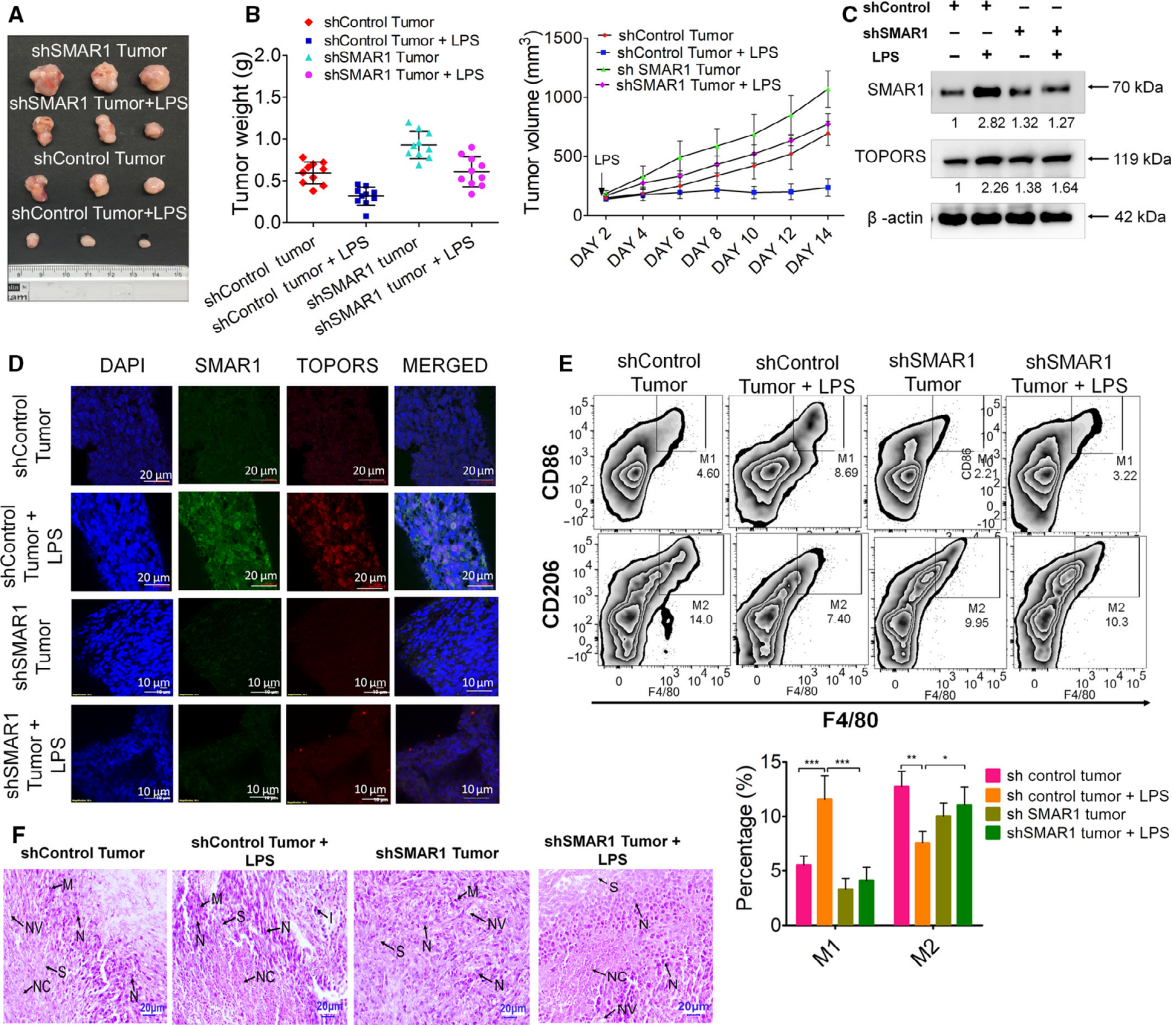
LPS reduces *in vivo* tumor growth by regulating SMAR1‐mediated macrophage polarization. (A) Comparative tumor sizes of CT26 and CT26shSMAR1 tumors treated with either LPS (5 μg/100 μL) or PBS (100 μL; *n* = 15/group). (B) Graphical representation of tumor progression at different days post‐LPS treatment. (C) Western blot showing the expression of SMAR1 in tumor lysates. (D) Representative images of IHC study showing the expression of SMAR1 and TOPORS in tumor tissue upon LPS treatment. The scale bar represents 20 μm for shControl and 10 μm for *shSMAR1* tumor tissue, respectively. (E) Representative FACS plots showing the percentage of TAM polarization in the presence and absence of LPS injection in tumor‐bearing mice (F) Representative images of H& E staining of tumor tissue section showing the morphological differences upon LPS treatment [neoplastic cells (N), necrosis (NC), mitotic figures (M), neovascularization (NV), inflammation (I), and stroma (S)]. The scale bar represents 20 μm. β‐Actin was used as an endogenous loading control in all the western blots. The values below the blot represent the fold change relative to control, which was calculated after normalization with β‐actin. Data shown are representative of three independent experiments. Error bars indicate that all the values are mean ± SD, where *P < 0.05, **P < 0.01, and ***P < 0.001 (two‐way ANOVA, Bonferroni post‐tests).

As observed in the previous results, that SMAR1 dictates the differentiation of macrophages into M1 phenotype by suppressing STAT3 expression. Next, this phenomenon was investigated in *in vivo* tumor model. To establish an active TLR4 signaling within the host, the CT26 and CT26sh*SMAR1* cells were pretreated with LPS before injecting into the mice. We observed that LPS treatment drastically decreased the tumor burden. But it failed to show the same response in *SMAR1*‐silenced conditions (Fig. 5A). The tumor weight and volume were observed more in the untreated group with respect to the LPS‐treated group, while in *SMAR1‐*silenced condition, even LPS ‐treated mice displayed an enhanced tumor weight and volume (Fig. 5B). An enhanced expression of SMAR1 and TOPORS in tumors harvested from LPS‐treated mice was observed, but *SMAR1*‐silenced tumors were irresponsive to LPS treatment (Fig. 5C). This suggests that LPS do play an important role in tumor regression by inducing SMAR1. An IHC analysis of these tumor sections revealed that the tumors injected with LPS were displaying higher expression of SMAR1 and TOPORS as compared to the untreated set. On the contrary, LPS injection failed to replenish SMAR1 expression in *SMAR1*‐silenced tumors (Fig. 5D). To further delineate the *in vivo* tumor regression, the status of macrophages in the tumor microenvironment was analyzed. The homogenized tumor cells were stained with macrophage markers, that is, CD45R, F4/80 along with surface markers specific for M1(CD86) and M2(CD206) phenotype. FACS analyses revealed that tumors treated with LPS displayed 2.5% more M1 like profile as compared to the control group. However, M2 population dominated in the absence of SMAR1 irrespective of LPS treatment. (Fig. 5E). H&E staining of the tumor sections showed that LPS‐treated tumors exhibit more inflammatory regions and necrotic areas as compared to the control group (Fig. 5F). Hence, LPS‐induced SMAR1 skews the macrophage polarization toward anticancer M1 phenotype by regulating STAT3 expression.

## 4 Discussion

The ability of TLR4 ligands to remodulate the chromatin architecture for generating an antitumor immune response has led to numerous efforts for designing bacterial components based on cancer therapeutics. The current study highlights that LPS‐induced tumor regression, in part, occurs via regulation of tumor suppressor SMAR1 protein, which eventually dictates the macrophage polarization by reprogramming tumor‐associated macrophages (TAMs) to M1 phenotype via TLR4‐TRIF‐dependent pathway.

Several studies have been undertaken over the past few decades signifying the role of TLR4 ligands in eliciting antitumor immunity [53, 54, 55, 56]. Numerous reports suggest that *TLR4* mutations and its silencing result in tumor progression, which confirm that TLR4 signaling provides protection against cancer [40, 57, 58]. Different TLR ligands failed to enhance SMAR1 expression, whereas Poly(I:C) and LPS were able to induce SMAR1 expression. Precisely, TRIF‐dependent pathway is responsible for modulating SMAR1 expression, because inhibition of TLR4‐MyD88‐dependent signaling did not affect the potential of LPS to induce SMAR1. There are studies that support the fact that signaling via TLR4 is biased toward TRIF‐dependent route rather than MyD88 pathway [59, 60, 61]. Various previous reports also indicate that LPS stimulation favors the induction of TLR4‐MyD88‐independent gene expression [20].

*In silico* studies revealed that TOPORS was occupying *SMAR1* promoter region containing the palindromic consensus sequence [41]. Similar to SMAR1, TOPORS is also involved in chromatin modification and thereby refines the chromosomal architecture [30, 62]. ChIP analysis confirmed the presence of TOPORS on *SMAR1* promoter. Histone marks usually reflect whether a gene is transcriptionally active or inactive by virtue of its acetylation and methylation status. Histone H3 lysine 9 acetylation (H3K9Ac) mark denotes a transcriptionally active gene, whereas H3K27me3 is associated with transcriptional repression [63]. We observed an enhancement in H3K9Ac mark on *SMAR1* promoter upon LPS stimulation, which indicates that the gene is actively transcribing. Upon inhibiting TLR4 signaling, LPS treatment failed to increase the occupancy of TOPORS on *SMAR1* promoter, suggesting that TOPORS expression is also regulated by LPS. Like TOPORS, there are few other LPS‐inducible zinc finger proteins that are involved in different biological phenomena [64, 65]. Hence, it can be concluded that TOPORS acts as a potential transcription factor and helps in regulating the expression of tumor suppressor SMAR1 under LPS stimulation.

The adequate balance between STAT1 and STAT3 expression is very crucial for driving the macrophage polarization and tumor progression [66]. TLR4 ligands are already well known to skew the polarization of TAMs toward M1 phenotype and hence create an antitumor microenvironment [67]. SMAR1 is known to downregulate the transcription of *STAT3* in T cells [36]. We observed that LPS‐induced SMAR1 was able to regress the transcription of *STAT3* also in cancer cells. It has been seen that in the *SMAR1*‐silenced condition, LPS treatment was not effective enough to skew the macrophage polarization toward M1 phenotype. This indicates the importance of SMAR1 in maintaining the population of M1 macrophage.

**Fig. 6:**
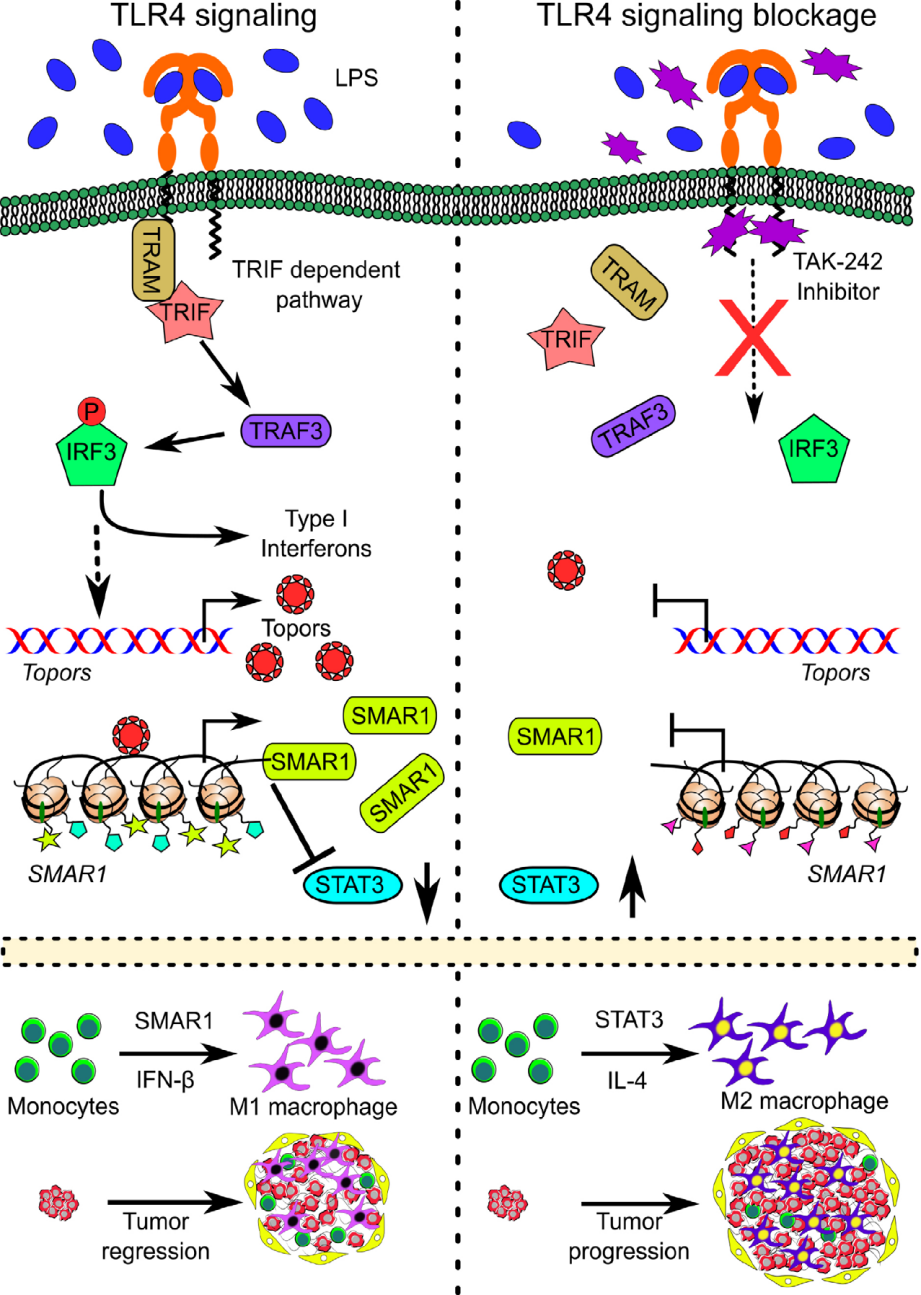
Working model showing the regulation of SMAR1 upon LPS treatment. TLR4 upon recognizing LPS initiates the signaling cascade. This leads to the activation of transcription factor IRF3 via TLR4‐TRIF‐dependent pathway. IRF3 upon phosphorylation translocates to the nucleus, where it regulates the expression of various type I interferons including IFN‐β. It could also induce the expression of zinc finger protein TOPORS, which further controls the transcription of tumor suppressor *SMAR1* via binding to the consensus region present on its promoter. LPS‐induced SMAR1 in return represses the transcription of *STAT3* and hence shifts the TAMs toward antitumor M1 profile, whereas upon inhibiting TLR4 pathway using a chemical inhibitor (TAK‐242), the downstream adapter proteins fail to bind to the TIR domain of TLR4. Under such circumstances, even LPS stimulation fails to enhance the expression of TOPORS and SMAR1. In the absence of effective SMAR1 concentration, the repression on *STAT3* promoter is released. In excess STAT3 conditions, most of the TAMs tend to polarize to M2 phenotype, which is associated with cancer growth and progression.

Altogether, our study revealed the molecular mechanism behind LPS‐mediated chromatin modification that regulates the expression of tumor suppressor SMAR1 (Fig. 6). Therefore, TLR4 ligands such as LPS and other modified chemical ligands [68] could be used to modify the global chromatin architecture in the tumor microenvironment and could regulate the expression of potential tumor suppressors in order to improve the overall clinical outcomes in cancer patients.

## 5 Conclusion

Our study demonstrated a novel molecular mechanism behind LPS‐mediated tumor regression that involves the transcriptional regulation of tumor suppressor *SMAR1* by TOPORS, a zinc finger binding transcription factor via TLR4‐TRIF axis.

## Conflict of interest

The authors declare no conflict of interest.

## Author contributions

PF, VKS, and SC conceptualized, planned, and designed the project. PF, VKS, and RP performed the experiments and acquired the data. PF, VKS, and SC analyzed the data. PF wrote the manuscript. PF, VKS, RP, and SC provided intellectual inputs and helped in preparing the manuscript.

### Peer Review

The peer review history for this article is available at https://publons.com/publon/10.1002/1878‐0261.13126.

## Supporting information


**Fig. S1**. SMAR1 is induced in a time‐dependent manner upon LPS stimulation.

**Fig. S2**. LPS induction triggers TLR4 internalizes to initiate TRIF signaling.

**Fig. S3**. LPS enhances TOPORS occupancy on *SMAR1* promoter.

**Fig. S4**. SMAR1 has an inverse correlation with STAT3.

**Table S1**. List of antibodies used for western blot and IHC/Confocal studies.
**Table S2**. List of primer sets used for quantitative real time PCR studies for determining the transcript levels of various genes.

## Data Availability

The data that support the findings of this study are available from the corresponding author upon reasonable request.
