# Supplementary figures and images for: RING finger protein TOPORS modulates the expression of tumor suppressor SMAR1 in colorectal cancer via the TLR4‐TRIF pathway
Source: Mol Oncol. 2022 Feb 5;16(7):1523–40. doi: 10.1002/1878-0261.13126 (PMC8978522; doi:10.1002/1878-0261.13126)

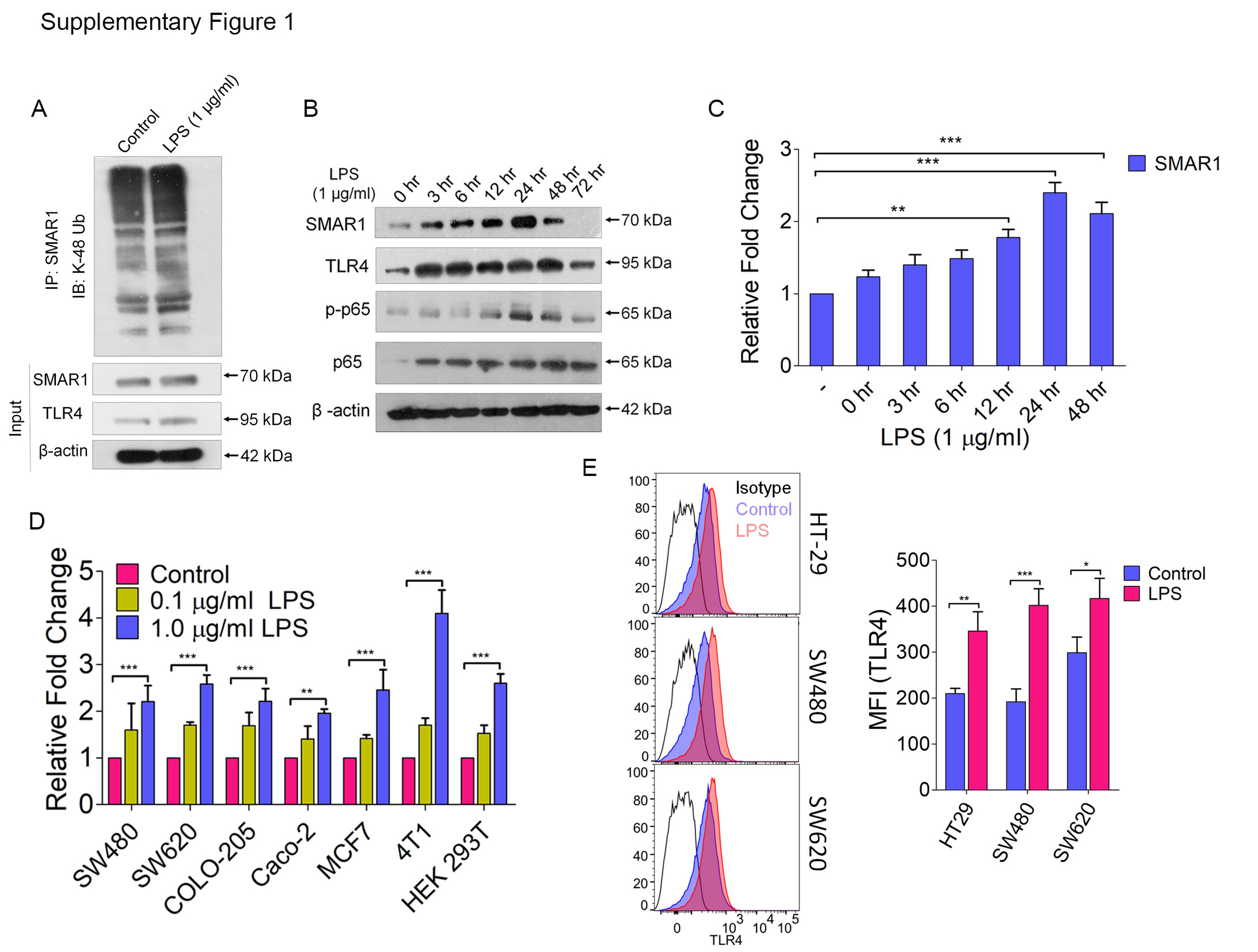

Supplement: Supplementary file 1 — Fig. S1. SMAR1 is induced in a time‐dependent manner upon LPS stimulation. [file MOL2-16-1523-s001.jpg]

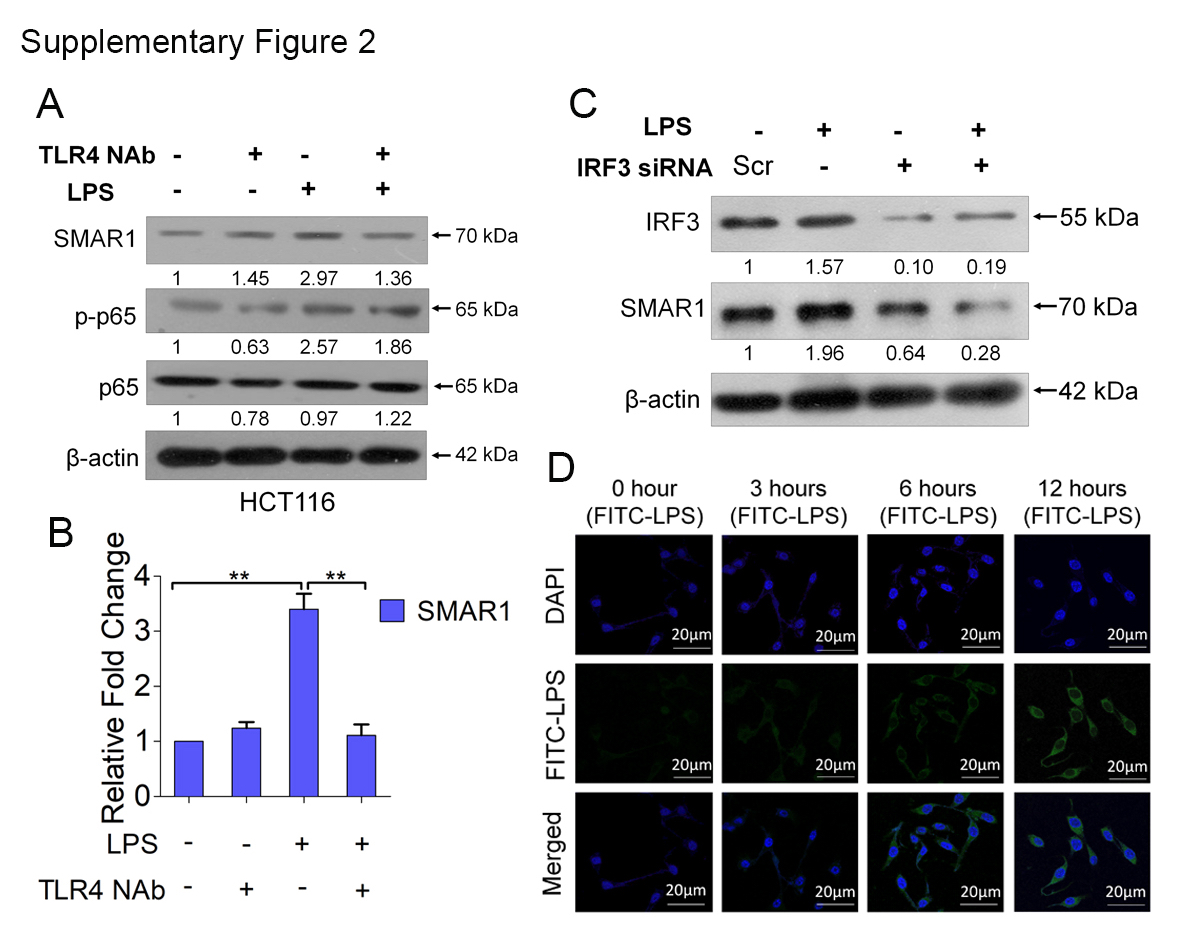

Supplement: Supplementary file 2 — Fig. S2. LPS induction triggers TLR4 internalizes to initiate TRIF signaling. [file MOL2-16-1523-s004.jpg]

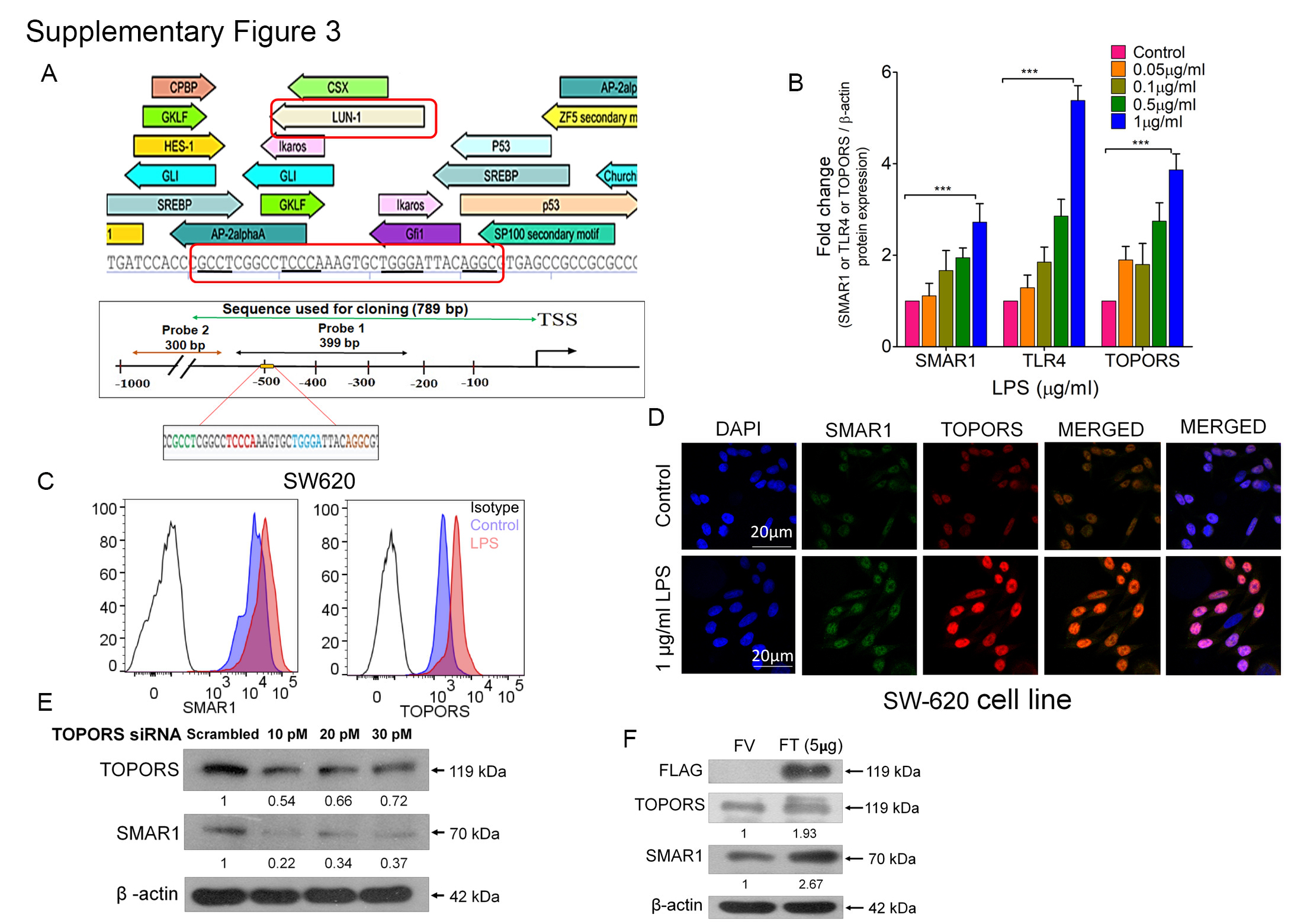

Supplement: Supplementary file 3 — Fig. S3. LPS enhances TOPORS occupancy on SMAR1 promoter. [file MOL2-16-1523-s003.jpg]

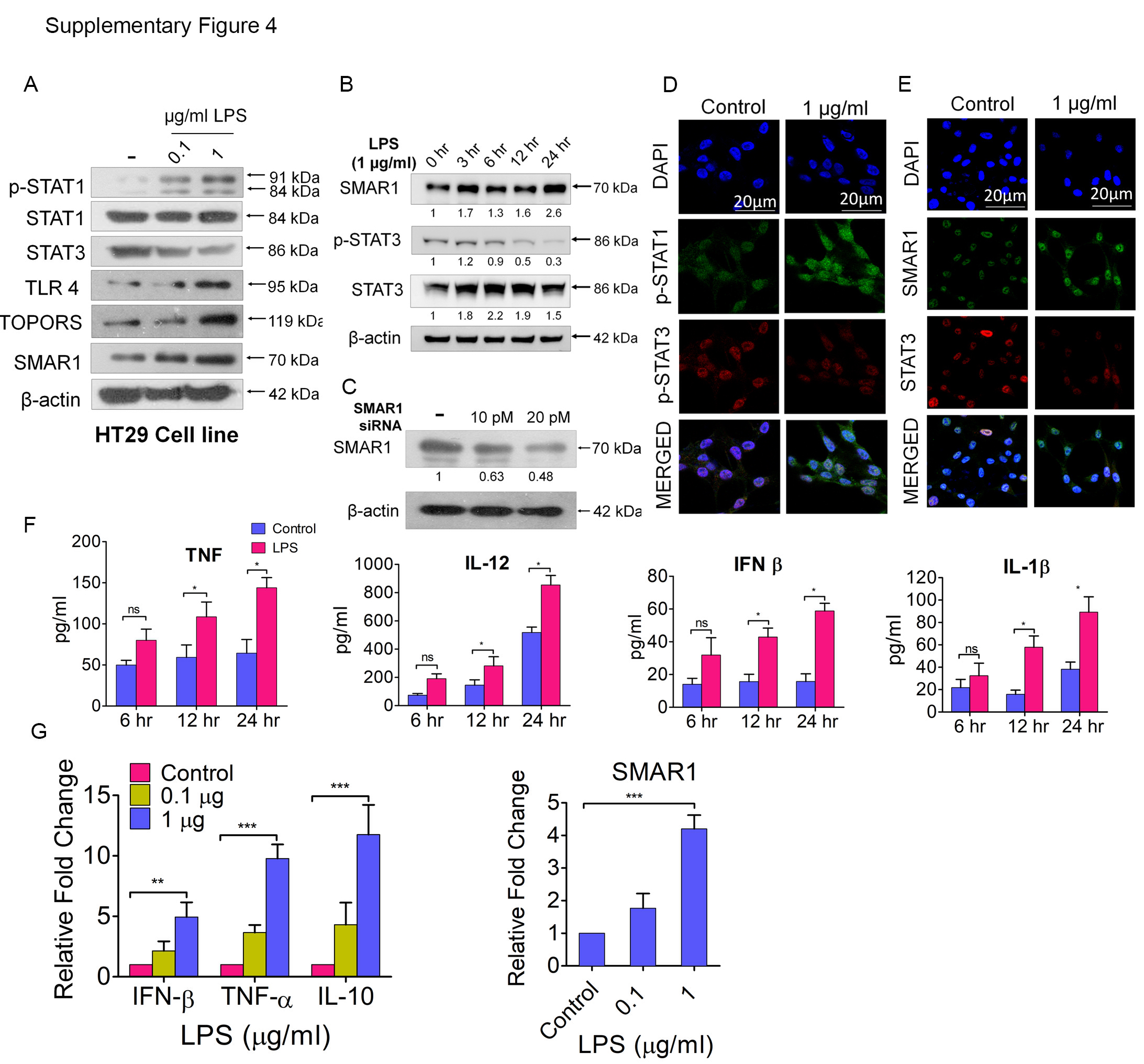

Supplement: Supplementary file 4 — Fig. S4. SMAR1 has an inverse correlation with STAT3. [file MOL2-16-1523-s005.jpg]
