# Supplementary material for: RING finger protein TOPORS modulates the expression of tumor suppressor SMAR1 in colorectal cancer via the TLR4‐TRIF pathway
Source: Mol Oncol. 2022 Feb 5;16(7):1523–40. doi: 10.1002/1878-0261.13126 (PMC8978522; doi:10.1002/1878-0261.13126)
Supplement: Supplementary file 5 — Table S1. List of antibodies used for western blot and IHC/Confocal studies. Table S2. List of primer sets used for quantitative real time PCR studies for determining the transcript levels of various genes. [file MOL2-16-1523-s002.docx]

**RING finger protein TOPORS modulates the expression of tumor suppressor *SMAR1* in colorectal cancer via the TLR4-TRIF pathway**

**Priyanka Firmal^1^, Vibhuti Kumar Shah^1^, Richa Pant^1^ and Samit Chattopadhyay ^1,2,3*^**

1 National Centre for Cell Science, S.P. Pune University Campus, Ganeshkhind, Pune, 411007

2 Department of Biological Sciences, BITS Pilani, K. K. Birla Goa Campus; NH 17B, Zuarinagar, Goa, 403726

3 Indian Institute of Chemical Biology; 4, Raja S C Mullick Road, Jadavpur, Kolkata, 700032

*Corresponding author: [samitc@goa.bits-pilani.ac.in](mailto:samitc@goa.bits-pilani.ac.in) , [samitchatterji@yahoo.com](mailto:samitchatterji@yahoo.com)

**Running Title**: Tumor suppressor SMAR1 induction by TLR4

**Abbreviations**: Toll-like receptor 4 (TLR4), TIR-domain-containing adapter-inducing interferon-β (TRIF), Lipopolysaccharide (LPS), Really Interesting New Gene (RING), Scaffold/matrix attachment region-binding protein 1 (SMAR1), TOP1 Binding Arginine/Serine Rich Protein (TOPORS), Interferon regulatory transcription factor (IRF3), Signal transducer and activator of transcription (STAT), Tumor-associated macrophage (TAM), Colorectal cancer (CRC), Tumor-associated macrophages (TAMs), Transcription start site (TSS), Chromatin immunoprecipitation (ChIP), Interleukin (IL), Fluorescence-activated cell sorting (FACS)

**Keywords:** LPS, TLR4, TOPORS, SMAR1, STAT3, TAM, CRC

**Supplementary Figure 1. SMAR1 is induced in a time-dependent manner upon LPS stimulation.** Western blot showing (A) K-48 ubiquitination in LPS treated and control HCT116 (B) expression of SMAR1 in a time dependent manner upon LPS treatment. (C) Real time PCR showing a time dependent change in the levels of *SMAR1* transcript upon induction with LPS in HCT116 cell line. (D) Real time quantification of *SMAR1* transcript upon LPS treatment in various cancer cell lines. (E) FACS plots along with graph of respective Median Fluorescent Intensity to quantitate the expression of SMAR1 in different colon cancer cell lines. β-actin was used as an endogenous loading control in all the western blots. Relative fold change for all the real time quantitation was calculated using *18S rRNA*. Data shown are representative of three independent experiments. Error bars indicate that all the values are Mean ± S.D., where ** p<0.01 *** p<0.001 (One-way ANOVA, Bonferroni post-tests).

**Supplementary Figure 2. LPS induction triggers TLR4 internalizes to initiate TRIF signaling.** (A) Western blot analysis showing SMAR1 expression upon (A) inhibiting TLR4 pathway using Anti-TLR4 neutralizing antibody (HTA 125) in the absence and presence of LPS (1 μg/ml) treatment in HCT116 cell line. Only LPS treatment condition was used as positive control. (B) Real time analysis was carried out to check the transcript levels of *SMAR1* in HCT116 cell line upon inhibiting TLR4 pathway. (C) Western blot showing the expression of SMAR1 upon silencing *IRF-3* in CT26 cell line in the presence and absence of LPS. (D) Representative immunofluorescence images showing the uptake of FITC-LPS in a time dependent manner in HCT116 cell line. The scale bar represents 20 μm. β-actin was used as an endogenous loading control in all the western blots. The values below the blot represents the fold change relative to control, which was calculated after normalization with β-actin. Relative fold change for all the real time quantitation was calculated using *18S rRNA.* Data shown are representative of three independent experiments. Error bars indicate that all the values are Mean ± S.D., **p<0.01(One-way ANOVA, Bonferroni post-tests).

**Supplementary Figure 3. LPS enhances TOPORS occupancy on *SMAR1* promoter.** (A) In silico analysis of *SMAR1* promoter using TRANSFAC database displaying a potential TOPORS binding site. (B) Representative western blot quantitation graph showing the expression of SMAR1, TLR4 and TOPORS in LPS treated condition with respect to control. (C) FACS plot depicting the expression of SMAR1 and TOPORS in SW620 cell line upon LPS treatment. (D) Representative immunofluorescence images showing the expression of SMAR1 and TOPORS in SW620 cell line upon LPS treatment. Western blot displaying (E) the silencing of *TOPORS* using siRNA and (F) expression of SMAR1 upon TOPORS overexpression in HCT116 cell line. The scale bar represents 20 μm. β-actin was used as an endogenous loading control in all the western blots. The values below the blot represents the fold change relative to control, which was calculated after normalization with β-actin. Relative fold change for all the real time quantitation was calculated using *18S rRNA*. Data shown are representative of three independent experiments. Error bars indicate that all the values are Mean ± S.D., **p<0.01(One-way ANOVA, Bonferroni post-tests).

**Supplementary Figure 4. SMAR1 has an inverse correlation with STAT3*.*** (A) Western blot showing the relationship between SMAR1 and STAT3 expression pattern in HT29 cell line in the presence of LPS. Western blot analysis to determining the expression of STAT3 and SMAR1 under LPS treatment (1µg/ml) by (B) a time dependent western blot analysis in CT26 cell line. (C) Western blot displaying the expression of SMAR1 upon siRNA treatment. (D) Confocal images showing the expression of pSTAT1 and pSTAT3 as well as (E) STAT3 and SMAR1 in CT26 cell line upon LPS treatment. (F) ELISA for quantitating the concentration of proinflammatory cytokine production in the CM of CT26 cells upon LPS treatment at 6, 12 & 24 hours. (G) Real time PCR showing the transcript levels of different proinflammatory cytokines and *SMAR1* in peritoneal macrophage (n=6) under dose dependent LPS treatment. The scale bar represents 20 μm. β-actin was used as an endogenous loading control in all the western blots. The values below the blot represents the fold change relative to control, which was calculated after normalization with β-actin. Relative fold change for all the real time quantitation was calculated using *18S* *rRNA*. Data shown are representative of three independent experiments. Error bar indicates that all the values are Mean ± S.D., where * p<0.05, ** p<0.01, *** p<0.001 and ns: non-significant.

**Supplementary Table 1:** List of antibodies used for western blot and IHC/Confocal studies.

| Antibody | Supplier | Cat. no. | Dilution  (Western blot) | Dilution  (IHC/Confocal) |
| --- | --- | --- | --- | --- |
| Rabbit polyclonal  anti-SMAR1 | Bethyl Laboratories | A300-279A | 1:4000 | 1:500 |
| Mouse monoclonal  anti-TOPORS  (Used for cell lines of human origin) | Santa Cruz Biotechnology | sc-101182 | 1:2000 | 1:200 |
| Mouse monoclonal  anti-TOPORS  (Used for cell lines of mouse origin) | Novus Biologicals | H00010210-M01 | 1:2000 | 1:200 |
| Mouse monoclonal  anti-TLR4 | Santa Cruz Biotechnology | sc-293072 | 1:1000 | 1:200 |
| Goat polyclonal  anti-β-actin | Santa Cruz Biotechnology | sc-1615 | 1:2000 |  |
| Rabbit monoclonal  anti-p65 | Cell Signaling Technology | 8242S | 1:1000 |  |
| Rabbit monoclonal  anti-Phospho-p65 | Cell Signaling Technology | 3033S | 1:1000 |  |
| Rabbit polyclonal  anti-STAT1 | Cell Signaling Technology | 9172S | 1:1000 | 1:100 |
| Rabbit monoclonal  anti-pSTAT1 | Cell Signaling Technology | 7649S | 1:1000 | 1:100 |
| Rabbit monoclonal  anti-STAT3 | Cell Signaling Technology | 12640S | 1:1000 | 1:100 |
| Rabbit monoclonal  anti-pSTAT3 | Cell Signaling Technology | 9134S | 1:1000 | 1:100 |
| Rabbit monoclonal  anti-IRF3 | Cell Signaling Technology | 4302S | 1:1000 | 1:100 |
| Rabbit monoclonal  anti-pIRF3 | Cell Signaling Technology | 29047S | 1:1000 |  |
| Rabbit polyclonal  anti-JNK | Cell Signaling Technology | 9252S | 1:1000 |  |
| Rabbit monoclonal  anti-pJNK | Cell Signaling Technology | 4668S | 1:1000 |  |
| Rabbit monoclonal  anti-FLAG | Cell Signaling Technology | 14793S | 1:1000 |  |
| Mouse monoclonal  anti-p53 | Santa Cruz Biotechnology | sc-126 | 1:1000 |  |

**Supplementary Table 2:** List of primer sets used for quantitative real time PCR studies for determining the transcript levels of various genes.

| S.No. | Primer | Sequence (5’-3’) |
| --- | --- | --- |
| 1 | h*SMAR1* Fwd | CTTGCGGTTGGATAGCATTGA |
| 2 | h*SMAR1* Rev | GCTGCTTGTTCGTGACCAGAT |
| 3 | 18S *rRNA* Fwd | GGCCCTGTAATTGGAATGAGTC |
| 4 | 18S *rRNA* Rev | CCAAGATCCAACTACGAGCTT |
| 5 | m*IL-10* Fwd | CGGGAAGACAATAACTGCAC |
| 6 | m*IL-10* Rev | CATTTCCGATAAGGCTTGG |
| 7 | m*IFN-β* Fwd | TTACACTGCCTTTGCCATCC |
| 8 | m*IFN-β* Rev | ACTGTCTGCTGGTGGAGTTCAT |
| 9 | m*TNF-α* Fwd | CACGTCGTAGCAAACCACC |
| 10 | m*TNF-α* Rev | TGGGAGTAGACAAGGTACAACC |
| 11 | m*SMAR1* Fwd | ACGCCATCCTCATCCTCTTA |
| 12 | m*SMAR1* Rev | GGGATTACTTGCACCTGTCC |
| 13 | *TOPORS* OE Fwd | CCGGAATTCATGGCATCAGCTGCTAAGG |
| 14 | *TOPORS* OE Rev | GGCGGTACCGGCAGTTTTAAGACATATCACAG |
| 15 | *SMAR1* Pro Fwd | TTATTGGCAAAAGGGAGTTGGG |
| 16 | *SMAR1* Pro Rev | CGAGGCAGCTATTTTCACTGG |
